# SCF/C-Kit/JNK/AP-1 Signaling Pathway Promotes Claudin-3 Expression in Colonic Epithelium and Colorectal Carcinoma

**DOI:** 10.3390/ijms18040765

**Published:** 2017-04-06

**Authors:** Yaxi Wang, Tingyi Sun, Haimei Sun, Shu Yang, Dandan Li, Deshan Zhou

**Affiliations:** 1 Department of Histology and Embryology, School of Basic Medical Sciences, Capital Medical University, Beijing 100069, China; wangyaxi1@sina.com (Y.W.); styj211@ccmu.edu.cn (T.S.); haimei@ccmu.edu.cn (H.S.); sheilayslamb@aliyun.com (S.Y.); lidan0135@sina.com (D.L.); 2 Beijing Key Laboratory of Cancer Invasion and Metastasis Research, Beijing 100069, China; 3 Cancer Institute of Capital Medical University, Beijing 100069, China

**Keywords:** claudins, colorectal cancer, c-kit, claudin-3, c-Jun N-terminal kinase, activator protein-1

## Abstract

Claudin-3 is a major protein of tight junctions (TJs) in the intestinal epithelium and is critical for maintaining cell-cell adhesion, barrier function, and epithelium polarity. Recent studies have shown high claudin-3 levels in several solid tumors, but the regulation mechanism of claudin-3 expression remains poorly understood. In the present study, colorectal cancer (CRC) tissues, HT-29 and DLD-1 CRC cell lines, CRC murine model (C57BL/6 mice) and *c-kit* loss-of-function mutant mice were used. We demonstrated that elevated claudin-3 levels were positively correlated with highly expressed c-kit in CRC tissues based upon analysis of protein expression. In vitro, claudin-3 expression was clearly increased in CRC cells by overexpressed c-kit or stimulated by exogenous recombinant human stem cell factor (rhSCF), while significantly decreased by the treatment with c-kit or c-Jun N-terminal kinase (JNK) inhibitors. Chromatin immunoprecipitation (ChIP) and luciferase reporter assay showed that SCF/c-kit signaling significantly promoted activator protein-1 (AP-1) binding with *CLDN-3* promoter and enhanced its transcription activity. Furthermore, decreased expression of claudin-3 was obtained in the colonic epithelium from the *c-Kit* loss-of-function mutant mice. In conclusion, SCF/c-kit-JNK/AP-1 signaling pathway significantly promoted claudin-3 expression in colonic epithelium and CRC, which could contribute to epithelial barrier function maintenance and to CRC development.

## 1. Introduction

Claudins are the major components of tight junctions (TJs) in the epithelium, and 27 family members widely distribute in different tissues. It is well known that claudins play important roles in maintaining cell-cell adhesion, barrier function, and epithelial physiological polarity [1,2]. Disruption of claudins frequently leads to anomalous structure and dysfunction of TJs, which may cause barrier related diseases [3]. Among the claudin members, claudin-3 is mainly expressed in basolateral membrane and the apical of the epithelium and is a main protein of TJs in the intestinal epithelium. Its absence often results in leaky paracellular permeability, which plays an important role in the pathogenesis of inflammatory bowel diseases [4]. In recent years, it has been reported that claudin-3 was highly expressed in ovarian cancers, but hardly detected in normal ovarian epithelium [5], thereby, claudin-3 was considered to be a promising target for diagnosis and treatment in ovarian cancers [6]. Furthermore, claudin-3 was also overexpressed in breast [7] and prostate [8] cancers. In fact, claudin members are often highly expressed in a variety of gastrointestinal tumors, for example, claudin-3, -4, and -7 are highly expressed in early esophageal adenocarcinoma [9], claudin-1 is highly expressed in gastric cancer [10], and claudin-1 and -2 are highly expressed in colorectal cancer (CRC) [11]. These results indicate that abnormally elevated claudins are positively correlated with tumorigenesis and progression of gastrointestinal tumors [12]. However, the molecular mechanism underlying the regulation of claudins expression remains poorly understood.

Recent studies have suggested that the activation of signaling molecules belonging to the receptor tyrosine kinase (RTK) superfamily are involved in up-regulating claudins expression during inflammation and several cancers. For instance, epidermal growth factor (EGF)/epidermal growth factor receptor (EGFR) has been reported to be responsible for elevated claudin-2 expression in CRC [13] and lung cancer [14]. It was further found that the activation of mitogen-activated protein kinase (MAPK), the downstream hub molecules of RTKs, was common in the abnormal expression of claudins [15,16,17]. It is worth mentioning that aberrant activation of c-kit, another RTK superfamily member and its ligand, stem cell factor (SCF), signaling was considered to play key roles in tumor cell proliferation, migration and invasion, which contributed to the aggressive biological behaviors of CRC and the shortened survival period of CRC patients [18]. Noticeably, claudin-3 was also overexpressed in these patients [19], implying a possibility that SCF/c-kit signaling might be a potential factor in the regulation of claudin-3 expression in colonic epithelium and CRC.

Therefore, the main aim of this study is to investigate the detailed mechanism of claudin-3 expression regulated by SCF/c-kit signaling. Our results indicated that high expression of claudin-3 was positively correlated with the activation of SCF/c-kit signaling. Furthermore, activation of c-Jun N-terminal kinase (JNK) and activator protein-1 (AP-1) molecules were found to be able to effectively promote claudin-3 expression in CRC cells. These results provide insight into the molecular mechanisms underlying the regulation of claudin-3 expression in the intestinal epithelium and in several tumor tissues.

## 2. Results

### 2.1. Clauin-3 Level Is Positively Correlated with C-Kit Expression in Colorectal Cancer (CRC) Tissues

**Figure 1:**
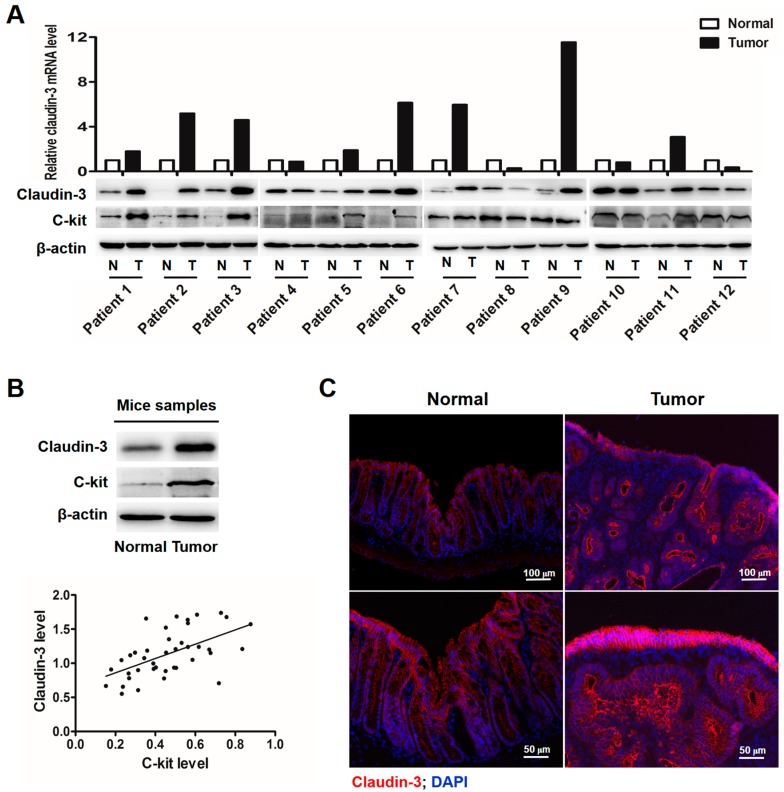
Claudin-3 and c-kit expressions are positively correlated in colorectal cancer (CRC) tissues. (**A**) Real-time polymerase chain reaction (PCR) was performed to detect *CLDN-3* mRNA level in CRC tissues and adjacent normal tissues from 12 patients. *Glyceraldehyde-3-phosphate dehydrogenase* (*GAPDH*) was used as the internal control. Normalized *CLDN-3* mRNA expression was shown in the column graph (N, normal; T, tumor). Western blot showing the protein expression of claudin-3 which was consistent with its mRNA level; (**B**) Western blot revealed that c-kit and claudin-3 were both highly expressed in CRC tissues from mice. β-Actin was used as the loading control. A significant positive correlation between c-kit and claudin-3 protein expressions was observed by Spearman correlation analysis in mouse CRC tissues (*n* = 20, *r* = 0.6, *p* < 0.01); (**C**) Immunofluorescence staining showed elevated claudin-3 expression in mouse CRC tissues.

Previous studies suggested that activation of c-kit signaling [20] and overexpression of claudin-3 [21] were both involved in CRC development. To clarify the correlation between c-kit and claudin-3, we collected 12 tumor samples from CRC patients and matched adjacent normal tissues. Claudin-3 mRNA and protein levels in CRC tissues were significantly elevated in 8 out of 12 patients. Simultaneously, c-kit protein was also shown to be up-regulated in these eight CRC samples (Figure 1A). The same phenomenon was also seen in mice CRC, and there was a significant positive correlation between c-kit and claudin-3 protein expressions (*r* = 0.6, *p* < 0.01; Figure 1B). Furthermore, immunofluorescence staining showed a high expression of claudin-3 in mouse CRC tissues compared to the normal tissues (Figure 1C).

### 2.2. Stem Cell Factor (SCF)/C-Kit Signaling Significantly Increases Claudin-3 Expression

**Figure 2:**
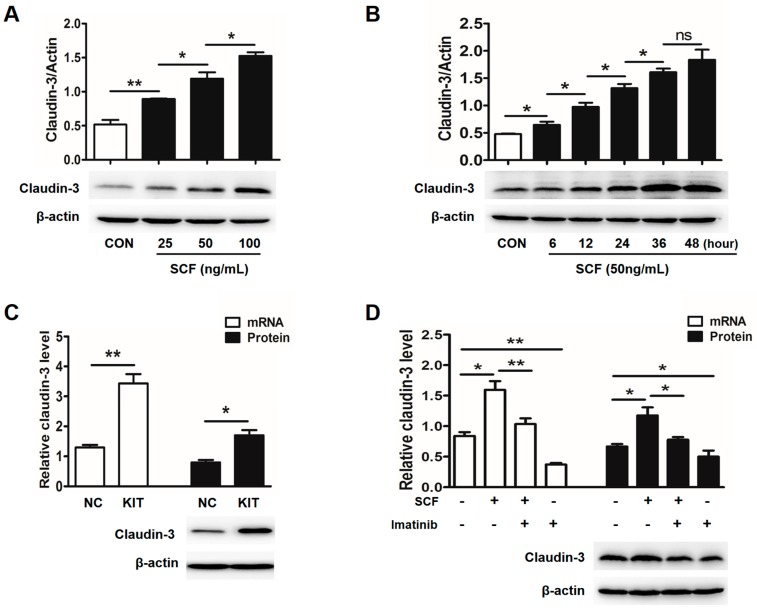
Stem cell factor (SCF)/c-kit signaling clearly increases claudin-3 expression in HT-29 cells. Activated SCF/c-kit signaling by exogenous rhSCF in HT-29 cells increased claudin-3 expression in a dose- (**A**) and time- (**B**) dependent manner (* *p* < 0.05, ** *p* < 0.01, ns *p* > 0.05, Con, control; SCF, stem cell factor); (**C**) Lentivirus-mediated overexpression of c-kit clearly promoted claudin-3 mRNA and protein expressions (NC, lentivirus-control; KIT, lentivirus-c-kit); (**D**) HT-29 cells were incubated in the absence and presence of rhSCF (50 ng/mL), Imatinib (2 µM), or rhSCF plus Imatinib for 36 h. It was clearly seen that Imatinib treatment reduced the basal and rhSCF-induced claudin-3 mRNA and protein expressions. All the values are mean ± SEM of three independent experiments (* *p* < 0.05, ** *p* < 0.01, ns *p* > 0.05, Con, control).

To clarify the role of SCF/c-kit signaling, claudin-3 expression was examined in HT-29 cells which highly expressed c-kit. As expected, significantly increased expressions of claudin-3 protein were observed in a dose- and time-dependent manner after rhSCF administration (Figure 2A,B). Likewise, overexpression of c-kit via lentivirus mediation in HT-29 cells significantly increased claudin-3 mRNA and protein level (Figure 2C). While Imatinib, a specific tyrosine kinase inhibitor, clearly down-regulated the basal and rhSCF-induced high expression of claudin-3. The protein expression was consistent with their mRNA level (Figure 2D).

**Figure 3:**
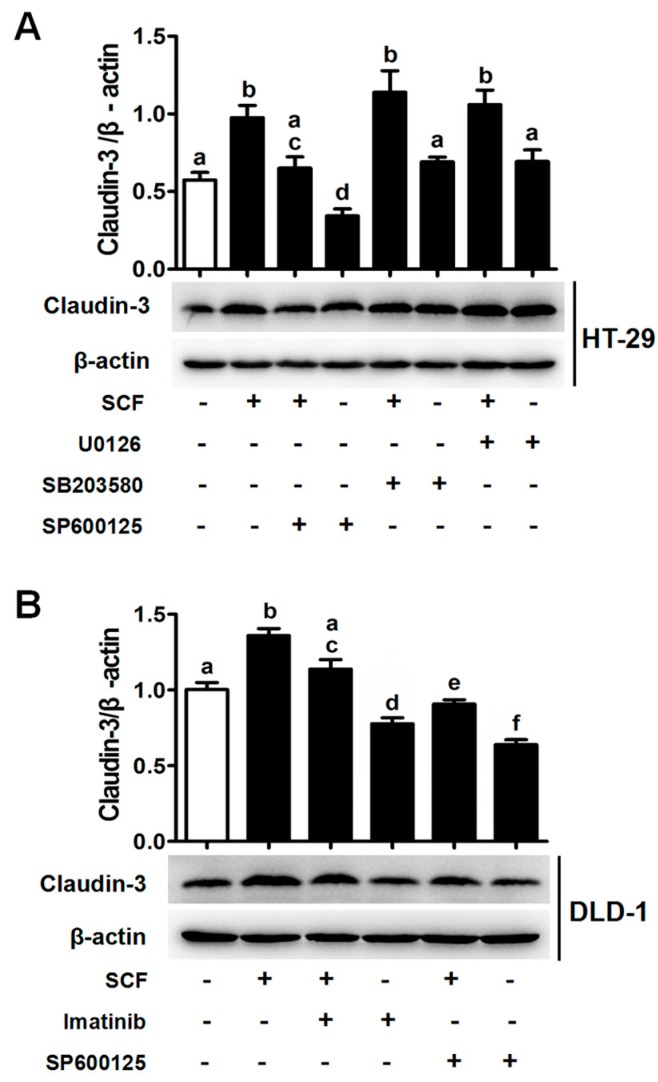
SCF/c-kit signaling increases claudin-3 expression via the c-Jun N-terminal kinase (JNK) pathway. (**A**) HT-29 cells were exposed to rhSCF alone or in combination with U0126 (ERK inhibitor), SB203580 (p38 inhibitor), or SP600125 (JNK inhibitor), respectively. Only SP600125 treatment significantly abrogated the rhSCF-induced claudin-3 expression; (**B**) The same experiments were performed in DLD-1 cells. Claudin-3 expression was increased after rhSCF administration, while decreased by the treatment with c-kit or JNK inhibitors. All the values are mean ± SEM of three independent experiments (*n* = 3, different letters indicate significant differences between groups).

In view of the involvement of MAPK pathways in SCF/c-kit signaling, HT-29 cells were treated with specific inhibitor of each of the MAPK molecules. The results showed that although rhSCF administration activated all three MAPK molecules (Supplementary Figure S1), blockage of JNK, rather than p38 or extracellular signal-regulated kinases (ERK), significantly attenuated the claudin-3 expression in the basal and rhSCF-treated case (Figure 3A). Similar results were also obtained from DLD-1 cells (Figure 3B and Supplementary Figure S2). Collectively, our results suggested that SCF/c-kit-MAPK/JNK signaling pathway could promote claudin-3 expression in CRC cells.

### 2.3. Activator Protein-1 (AP-1) Is Activated by c-Jun N-Terminal Kinase (JNK) in CRC Tissues

**Figure 4:**
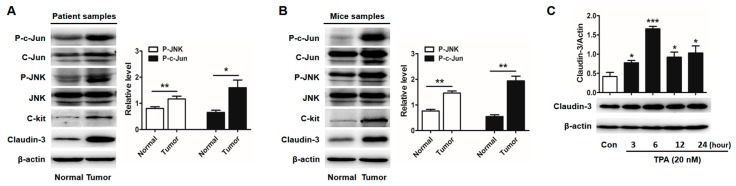
C-Jun mediates the c-kit/JNK-dependent increase in claudin-3 expression. Western blot indicated that p-JNK and p-c-Jun were increased in colorectal cancer (CRC) tissues of patients (**A**) and mice (**B**) in which both c-kit and claudin-3 were highly expressed. The levels of p-JNK and p-c-Jun were respectively represented relative to the value of JNK and c-Jun; (**C**) HT-29 cells were treated with phorbol 12-myristate 13-acetate (TPA) (20 nM) for different time periods and claudin-3 level was significantly elevated after TPA administration (TPA 3, 6, 12, 24 h vs. Con.). All the values are mean ± SEM of three independent experiments (* *p* < 0.05, ** *p* < 0.01, *** *p* < 0.001, Con., control).

Next, we searched the key transcriptional factors that could regulate *CLDN-3* transcription by using the on-line transcription factor prediction databases (JASPAR, http://jaspar.genereg.net/). We found that *CLDN-3* promoter region harbored the binding sites of AP-1/c-Jun, which was an important downstream transcription factor of JNK signaling. The transcriptional complex AP-1 consists of two subunits, c-Fos and c-Jun. Its activation predominantly depends on its dimer composition, Jun-Jun homodimers or Jun-Fos heterodimers. Thus, as the major component of AP-1, c-Jun phosphorylation plays an important role in AP-1 activation and is usually used as a marker of activated AP-1 [22]. We found that phosphorylation of JNK and c-Jun were both increased in CRC tissues which highly expressed c-kit and claudin-3 (Figure 4A,B). C-Jun phosphorylation was increased by rhSCF administration while decreased after pretreated with SP600125 in HT-29 cells and DLD-1 cells, consistent with the alterations of claudin-3 expression (Supplementary Figures S1 and S2). Furthermore, HT-29 cells treated with phorbol 12-myristate 13-acetate (TPA), a stimulator of AP-1, clearly elevated claudin-3 expression (Figure 4C).

### 2.4. AP-1/c-Jun Enhances CLDN-3 Promoter Activity

**Figure 5:**
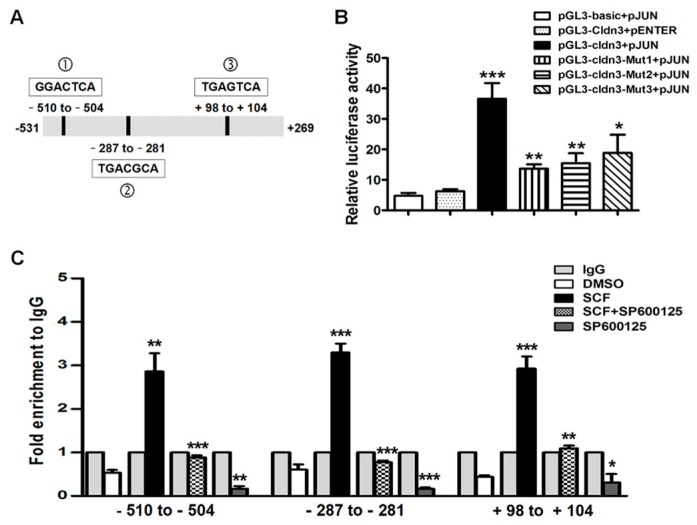
Binding ability of c-Jun to *CLDN-3* promoter is mediated by c-kit/JNK signaling pathway. (**A**) Three c-Jun binding sites were predicted in the *CLDN-3* promoter flanking region; (**B**) The dual luciferase reporter assays were performed at 24 h after transfection. C-Jun upregulated luciferase activity of *CLDN-3* promoter in 293T cells, but this role was abolished by mutating each binding site (pGL3-basic + pJun/pGL3-Cldn3 + pEnter vs. pGL3-Cldn3 + pJun, pGL3-Cldn3 + pJun vs. pGL3-Cldn3-Mut 1/2/3 + pJun); (**C**) HT-29 cells were treated with DMSO, rhSCF, SP600125, and rhSCF plus SP600125, respectively, followed by chromatin immunoprecipitation (ChIP) assays. Binding of c-Jun with the CLDN-3 promoter was effectively enhanced by activation of the SCF/c-kit/JNK signaling pathway. The values were shown relative to immunoglobulin G (IgG) which was set to 1 (SCF/SP600125 vs. DMSO, SCF + SP600125 vs. SCF). All the values are mean ± SEM of three independent experiments (* *p* < 0.05, ** *p* < 0.01, *** *p* < 0.001).

Three putative AP-1/c-Jun binding sites in the promoter region of the human *CLDN-3* gene were predicted (Figure 5A). To further identify the role of AP-1/c-Jun activation on claudin-3 expression, pGL3-Cldn3 luciferase reporter gene construct with c-Jun plasmid were co-transfected into HEK293T cells and the results are shown in Figure 5B. Over-expressed c-Jun obviously enhanced the luciferase activity of *CLDN-3* promoter, which was abrogated when mutating each c-Jun binding site. These results indicate that AP-1/c-Jun could increase *CLDN-3* transcription activity and that each of the three c-Jun binding sites was of great significance.

### 2.5. SCF/C-Kit/JNK Signaling Promotes the Binding of AP-1/c-Jun with CLDN-3 Promoter

Chromatin immunoprecipitation (ChIP) assays were performed to determine whether AP-1/c-Jun could bind the promoter region of *CLDN-3* with the anti-c-Jun antibody. Real-time PCR using three pairs of special primers against *CLDN-3* promoter showed that rhSCF induced a 5.5~7.3 fold enrichment of AP-1/c-Jun at each binding region compared with the control group. In contrast, markedly diminished occupancy was observed when the cell was treated with JNK inhibitor, SP600125 (Figure 5C). Clearly, AP-1/c-Jun could directly bind to the *CLDN-3* promoter, which was also influenced by c-kit/SCF/JNK signaling.

### 2.6. Claudin-3 Decreases in Colonic Mucosa of c-Kit Loss-of-Function Mutant Mice

**Figure 6:**
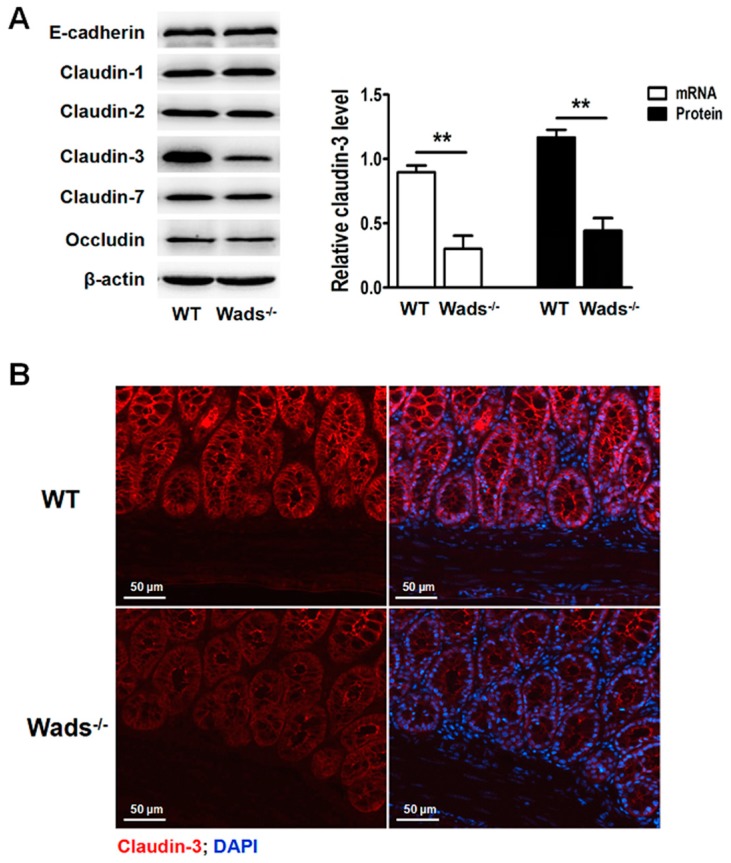
Claudin-3 is decreased in colonic epithelium of *c-Kit* loss-of-function mutant mice (Wads^−/−^). (**A**) Expressions of *E*-cadherin, claudin-1, -2, -3, -7, and occludin in the colonic epithelium of Wads^−/−^ mice and wild-type (WT) mice examined by western blot. Only claudin-3 significantly decreased in Wads^−/−^ mice compared to the WT mice. All the values are mean ± SEM of three independent experiments (*n* = 5, ** *p* < 0.01); (**B**) Claudin-3 expression was decreased in the colonic epithelium of Wads^−/−^ mice compared to the WT mice, as determined by immunofluorescence staining.

In the present study, *c-Kit* loss-of-function mutant mice (Wads^−/−^) were used for further examination of claudin-3 expression in vivo. Claudin-3 decreased in colonic mucosa, while other tight junction proteins, including claudin-1, -2, -7, occludin, and adhesion protein, *E*-cadherin, remained unchanged compared to the wild-type (WT) mice (Figure 6A). Similar results were obtained by immunofluorescence staining showing lower claudin-3 expression in colonic mucosa of the Wads^−/−^ mice (Figure 6B). These results further supported that SCF/c-kit signaling was crucial to maintain claudin-3 expression in the intestinal epithelium.

## 3. Discussion

TJs are polarized epithelial structures that are essential for keeping the epithelium more “tight” in organisms. Its breakdown leads to barrier dysfunction related diseases such as irritable bowel disease [4] and celiac disease [23]. Claudin-3 is an important protein of TJs and its absence impairs the blood-testis barrier during leptotene translocation [24]. Recent studies indicated potential functions of claudin-3 on tumorigenesis and revealed that high claudin-3 expression was correlated with shorter overall survival in several cancers [25]. In the present study, we observed that claudin-3 was obviously upregulated in the CRC tissues of patients and mouse models, which were consistent with other reports [26] in which high expression of claudin-3 was noted in CRC. However, information about the regulation of claudin-3 in the intestinal epithelium and tumor tissues is really limited. Previous studies suggested that highly-expressed claudin-3 could be induced by epigenetic processes including DNA methylation and histone H3 acetylation [27].

In our study, we found that highly expressed claudin-3 was often associated with the activation of SCF/c-kit signaling, suggesting that this signaling might play an important role in the regulation of claudin-3 expression in CRC. This hypothesis was further confirmed by using *c-Kit* loss-of-function mutant mice that had weaker claudin-3 expression in the colonic mucosa compared to the WT mice. Our results were consistent with the earlier reports that RTK signaling participated in regulating claudins expression. For example, EGF/EGFR widely modulated claudins expression in various organs including lung [14], kidney [28], and colon [13]; insulin-like growth factor 1 (IGF-1)/insulin-like growth factor 1 receptor (IGF-1R) was responsible for the change of claudin-1 expression during bone differentiation [29]; vascular endothelial growth factor (VEGF)/vascular endothelial growth factor receptor (VEGFR) was identified as an upstream regulator of claudin-5 expression [30]. Thus, RTK signaling clearly contributes to the regulation of claudins expression in multiple cells and tissues.

Regarding the molecular mechanism of claudins under the regulation by RTK signaling, it was considered that their common downstream molecules, MAPK, including three subfamilies, ERK1/2, p38, and JNK1/2/3, played a critical role. Ikari et al. [14] reported that PI-3 kinase and ERK1/2 pathways mediated claudin-2 expression in lung cancers. Whereas, in the current study, we identified that SCF/c-kit signaling increased claudin-3 expression only by activating the JNK pathway in colonic epithelium and CRC cells. This difference may be due to the different cells and tissues. It is well known that JNK often induces c-Jun phosphorylation, a subfraction of AP-1, at N-terminal Ser63 and Ser73, leading to the formation of activated AP-1 dimers. We found that the increased phosphorylation of JNK and c-Jun was often accompanied by highly expressed claudin-3 in colonic epithelium and CRC cells. It was well known that AP-1, as a multifunctional transcription factor, frequently regulates the expression of multiple genes related to cell proliferation, differentiation, and apoptosis [31]. Accordingly, we predicted three putative AP-1 binding sites in the promoter region of human *CLDN-3* gene and demonstrated that AP-1 could bind to the *CLDN-3* promoter region and that each of the three AP-1 binding sites significantly increase *CLDN-3* transcription activity. Moreover, the HT-29 cells treated with rhSCF, TPA, or corresponding inhibitors also elevated or reduced claudin-3 levels, further confirming the role of AP-1 in claudin-3 expression. Taken together, we considered that SCF/c-kit-JNK-AP-1 signaling axis played a critical role in the regulation of claudin-3 expression in colonic epithelium and CRC, which might contribute to maintaining epithelium barrier functions and body homeostasis, physiologically. However, the aberrant activation of these signal molecules would alter the claudin expression and function that are involved in the development of several solid tumors. It was reported that claudin-2 could form a complex with zonula occludens (ZO-1), ZO-1-associated protein (ZONAB), and cyclin D1 leading to enhancement of cell proliferation, which contributed to the development of lung cancer [32]. It enlightened us of the possibility that highly-expressed claudin-3 regulated by SCF/c-kit signaling might be involved in colorectal tumorigenesis. In addition, the interaction between the claudins with actin could alter cell movement [33], which might also be associated with tumor invasion and migration. Therefore, the detailed role and molecular mechanism of claudin-3 in CRC needs to be further explored.

## 4. Materials and Methods

### 4.1. Ethics Statement

All animals’ studies were carried out strictly under protocols approved by the Animal Care and Use Committee of Capital Medical University (Permit Number AEEI-2014-058, 17 June 2014). Every effort was made to minimize the number of animals used as well as their suffering.

### 4.2. Patient Samples

The clinical CRC samples, including 12 pairs of primary CRC tumors and their adjacent non-tumorous tissues, were collected immediately after surgical resection prior to any other therapeutic intervention at the Xuanwu Hospital Capital Medical University (Beijing, China). The study protocol was approved by the Clinical Research Ethics Committee of the Xuanwu Hospital Capital Medical University (Permit Number 2013-X-036, 20 July 2013). All patients were chemotherapy and radiation therapy naive and their samples were confirmed by pathological examination. The tissue samples were stored at −80 °C immediately after collection.

### 4.3. Establishment of CRC Murine Model

CRC models of C57BL/6 mice were established through azoxymethane (AOM, 10 mg/kg) (Sigma-Aldrich, St. Louis, MO, USA) injection and followed by three periods of 2.5% dextran sodium sulfate (DSS; MP Biomedicals, Solon, OH, USA) in drink water, as previously described [20]. The mice were sacrificed at 12, 20, 28, and 36 weeks after AOM injection, respectively. Half of the neoplasms were frozen in liquid nitrogen for protein and mRNA detections and the rest was fixed immediately in 4% paraformaldehyde for paraffin embedding or optimal cutting temperature (OCT) compound embedding. Sections were cut for immunofluorescence and/or hematoxylin-eosin (HE) staining.

### 4.4. RNA Exraction and Real-Time PCR

Total RNA was extracted from CRC tissues and cultured cells with TRIzol reagent (Life Technologies, Carlsbad, CA, USA). Reverse transcription reactions were performed using High-Capacity cDNA Reverse Transcription Kit (Life Technologies). Twenty microliter reactions were incubated in a Veriti 96-well Thermal Cycler (Life Technologies) for 40 min at 42 °C and 5 min at 85 °C. Real-time PCR was performed in an ABI 7500 real-time PCR system (Life Technologies) using Ultra SYBR Mixture with ROX (CWBiotech, Beijing, China). The following primers are listed in Supplementary Table S1. Twenty-five microliter reactions were incubated at 95 °C for 10 min, followed by 40 cycles at 95 °C for 10 s, 60 °C for 10 s, and 72 °C for 40 s. All PCR reactions were run in triplicate. Relative gene expression was determined using comparative CT (2-DeltaDeltaCt calculation, 2^−ΔΔ*C*t^) method.

### 4.5. Western Blot

Total protein was extracted from CRC tissues and cultured cells and subjected to immunoblotting, as previously described [20]. Primary antibodies including rabbit polyclonal anti-claudin-1 (1:1000), anti-claudin-2 (1:500), anti-claudin-3 (1:1000), and anti-claudin-7 (1:1000) were obtained from Abcam (Cambridge, MA, USA). Primary antibody rabbit polyclonal anti-occludin (1:400) was purchased from Invitrogen (Carlsbad, CA, USA). Primary antibodies including rabbit polyclonal anti-*E*-cadherin (1:1000), anti-c-kit (1:1000), anti-c-Jun (1:1000), anti-p-c-Jun (1:1000), anti-JNK (1:1000), and anti-p-JNK (1:1000) were obtained from Cell Signaling Technology (Beverly, MA, USA). Mouse monoclonal anti-β-actin (1:2000) was purchased from Santa Cruz Biotechnology (Santa Cruz, CA, USA). The proteins were detected using enhanced chemiluminescence (ECL) (ThermoFisher Scientific, Waltham, MA, USA) and viewed in Fusion FX Vilber Lourmat (Paris, France).

### 4.6. Immunofluorescence Staining

Cryosections (5 µm) were fixed with 4% paraformaldehyde for 20 min at 25 °C. Non-specific binding was blocked with 5% goat serum in phosphate-buffered saline containing 0.2% Triton X-100 and 1% bovine serum albumin (Sigma-Aldrich) for 1 h. After incubation with rabbit polyclonal antibodies against claudin-3 (1:400, Invitrogen) in 0.1% bovine serum albumin (BSA) overnight at 4 °C, the sections were washed and incubated with Cy3-conjugated goat anti-rabbit IgG antibody (1:400, Invitrogen) for 1 h at 25 °C. Images were obtained with a fluorescence microscope (Nikon 80i, Sendai, Japan).

### 4.7. Cell Culture

The human CRC cell lines, HT-29 and DLD-1 cells, and normal HEK293 cell line were purchased from American Type Culture Collection (ATCC, Manassas, VA, USA). All cells were cultured in Dulbecco's modified eagle’s medium (DMEM ) supplemented with 10% fetal bovine serum and 1% penicillin/streptomycin (Life Technologies) at 37 °C in the presence of 5% CO_2_.

The cells were treated with recombinant human SCF (rhSCF) (R&D System, Minneapolis, MN, USA) in a gradient of concentrations (0, 25, 50, 100 ng/mL) or for different time (0, 6, 12, 24, 36, 48 h) or phorbol 12-*O*-tetradecanoate-13-acetate (TPA, 20 nM; Sigma-Aldrich) for different times (3, 6, 12, 24 h). After serum starvation overnight, rhSCF (50 ng/mL) was added into the medium 1 h after specific inhibitor treatment including SP600125 (JNK inhibitor, 10 µM; Sigma-Aldrich), SB203580 (p38 inhibitor, 10 µM; Sigma-Aldrich) and U0126 (extracellular signal regulated kinase (ERK) inhibitor, 10 µM; Sigma-Aldrich), respectively. The cells were harvested 36 h later and used for further experiments.

### 4.8. Lentiviral Vector Construction and Infection

To perform lentivirus mediated overexpression of c-kit, HT-29 cells were seeded in a 6-well plate and infected with the c-kit lentiviral vector (GV287, GeneChem, Shanghai, China) when 30% confluency was reached. The infection efficiency was evaluated by the enhanced green fluorescent protein (EGFP) expression with an inverted florescence phase contrast microscope (Leica DMI3000 B, Brunswick, Germany).

### 4.9. Construction of Plasmids

The promoter region of the human *CLDN-3* gene was amplified by PCR and then subcloned into Bgl II and Hind III sites of pGL3-basic plasmid (Promega, Madison, WI, USA) upstream of a luciferase reporter gene. The mutant AP-1 binding site was generated using a QuickChange Lightning Site-Directed Mutagenesis Kit (Agilent Technologies, Santa Clara, CA, USA). The primers used in construction or mutation are listed in Supplementary Table S2. AP-1 plasmid was purchased from ViGene (Rockville, MD, USA).

### 4.10. Luciferase Reporter Assay

HEK293T cells seeded in 96-well plates were transfected with *CLDN-3* promoter mimics luciferase reporter plasmid, AP-1 vector, and Renilla luciferase expression vector (Promega) using Lipofectamin 2000 (Life Technologies). Luciferase activities were measured at 24 h after transfection using a Dual-Glo Luciferase Assay kit (Promega) and firefly luciferase activities were normalized to Renilla luciferase activities.

### 4.11. Chromatin Immunoprecipitation (ChIP)

HT-29 cells were exposed to DMSO (Sigma, USA), rhSCF, rhSCF plus SP600125, and SP600125, respectively and then treated with 1% formaldehyde to crosslink the protein to DNA. ChIP assay and real-time PCR were performed as previously described [34]. The primers are shown in Supplementary Table S3.

### 4.12. Animals

Wads^−/+^ mice on a C57BL/6 background were purchased from the Model Animal Research Center of Nanjing University (Nanjing, China) and maintained in the standard environment conditions. Wild type (WT) and Wads^−/−^ mice were obtained by mating Wads^−/+^ parents as previously described [20]. Mice were genotyped by their distinct differences in fur pigmentation: black for WT, piebaldism for Wads^−/+^, and white for Wads^−/−^ mice.

### 4.13. Statistics

Results were presented as the means ± SEM and analyzed using a student’s *t*-test or one-way ANOVA with the SPSS 23.0 software (IBM Corporation, New York, NY, USA). A *p*-value of 0.05 or less was considered statistically significant.

## 5. Conclusions

In conclusion, the present study clearly revealed that activation of SCF/c-kit/JNK/AP-1 signaling axis could obviously up-regulate claudin-3 expression in the colonic epithelium and colorectal carcinoma. Our results provide insight into the molecular mechanisms underlying the regulation of claudin-3 expression which will help us to develop specific drugs for the clinical treatment of high claudin-3 related diseases.

## Supplementary Materials

Supplementary materials can be found at www.mdpi.com/1422-0067/18/4/765/s1.

## Abbreviations

TJs	Tight junctions
CRC	Colorectal cancer
JNK	C-Jun N-terminal kinase
ChIP	Chromatin immunoprecipitation
AP-1	Activator protein-1
RTK	Receptor tyrosine kinases
EGFR	Epidermal growth factor receptor
MAPK	Mitogen-activated protein kinase
SCF	Stem cell factor
rhSCF	Recombinant human SCF
ERK	Extracellular signal-regulated kinases
IGF-1	Insulin-like growth factor 1
VEGFR	Vascular endothelial growth factor
