# Supplementary material for: SCF/C-Kit/JNK/AP-1 Signaling Pathway Promotes Claudin-3 Expression in Colonic Epithelium and Colorectal Carcinoma
Source: Int J Mol Sci. 2017 Apr 6;18(4):765. doi: 10.3390/ijms18040765 (PMC5412349; doi:10.3390/ijms18040765)
Supplement: Supplementary file 1 [file ijms-18-00765-s001.pdf]

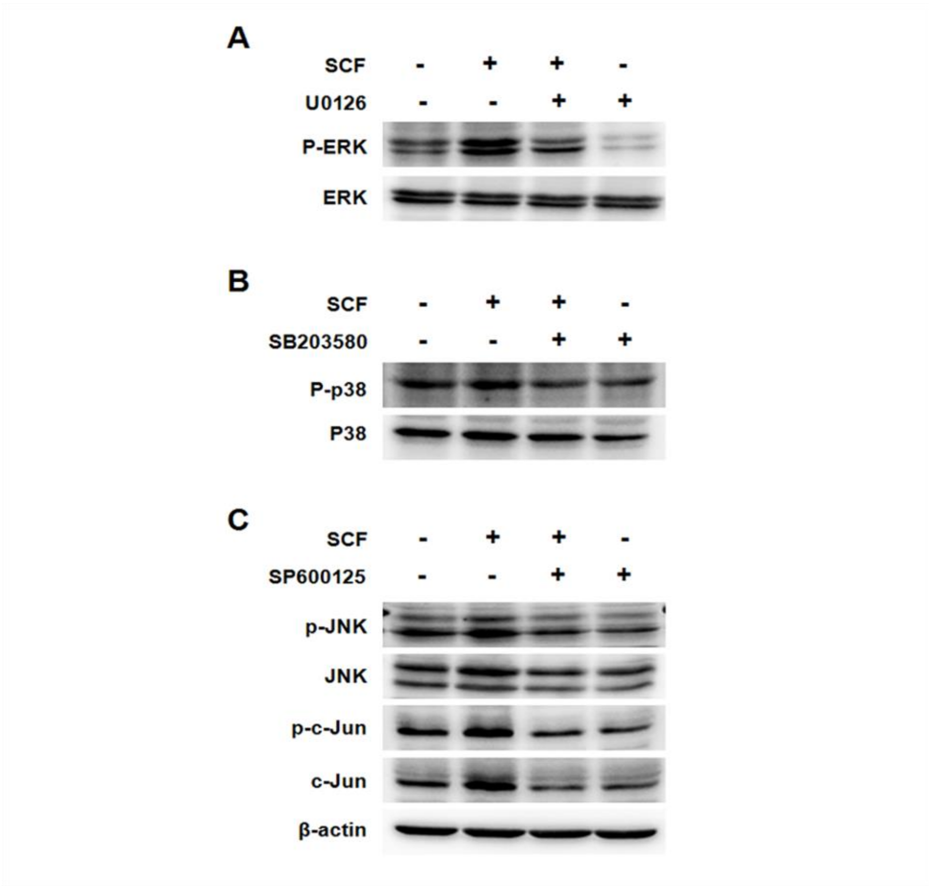

Supplementary Figure S1. HT-29 cells were exposed to rhSCF alone or in combination with U0126, SP600125, or SB203580, respectively. Phosphorylation of ERK1/2 (A), p38 (B) and JNK (C) was increased after rhSCF treatment while decreased after treatment with their inhibitors.

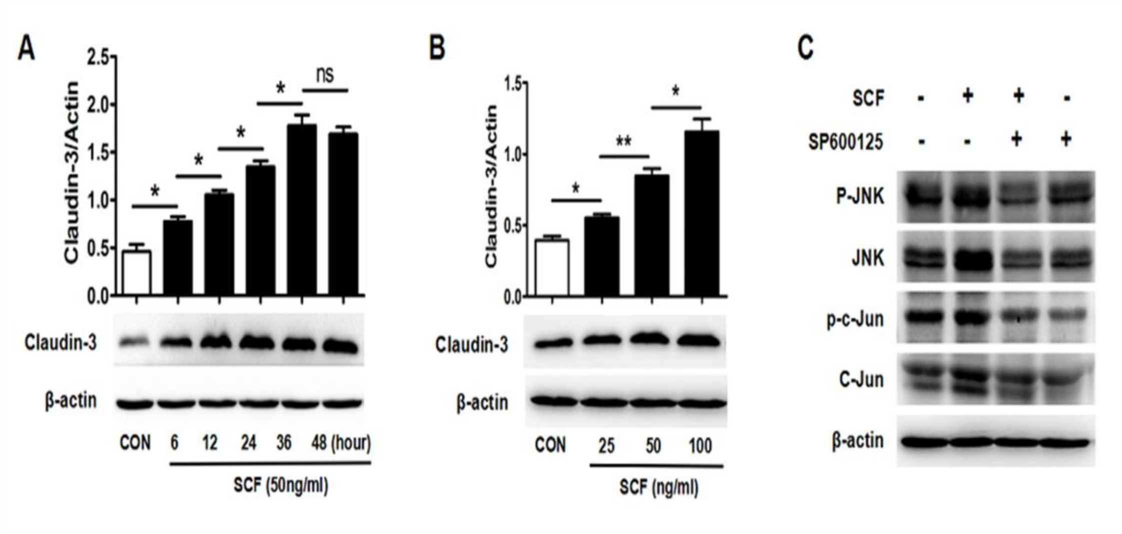

Supplementary Figure S2. Western blot indicated that activation of c-kit signaling by exogenous rhSCF in DLD-1 cells significantly increased claudin-3 expression in a time- (A) and dose- (B) dependent manner. The values are mean ± SEM of three independent experiments (* p<0.05, ** p<0.01); (C) C-Jun activated by SCF/c-kit signaling was attenuated by SP600125 treatment in DLD-1 cells.

Primer Name	Sequences
Cldn3	5′ - CCGCTCGAGGCATCTTTTGGGTACCTTTGCG - 3′
	5′ - GAAGATCTCCAGATGTTCTGCGACGTGATG - 3′
GAPDH	5′ - AACCCCACCGCCCGGTTACAGGCTGAGCCTGTG - 3′
	5′ - CACAGGCTCAGCCTGTAACCGGGCGGTGGGGTT - 3′

Supplementary Table S1. Primers for Real-time PCR

Primer Name	Sequences
Cldn3 promoter	5′ - CCGCTCGAGGCATCTTTTGGGTACCTTTGCG - 3′
	5′ - GAAGATCTCCAGATGTTCTGCGACGTGATG - 3′
Cldn3 Mutant 1	5′ - AACCCCACCGCCCGGTTACAGGCTGAGCCTGTG - 3′
	5′ - CACAGGCTCAGCCTGTAACCGGGCGGTGGGGTT - 3′
Cldn3 Mutant 2	5′ - CACCCCAAGCCCCTGTTACATCCCGTCCTCGGT - 3′
	5′ - ACCGAGGACGGGATGTAACAGGGGCTTGGGGTG - 3′
Cldn3 Mutant 3	5′ - GCGCGCCGTCGGTGTTACAGTCCGTCCGTCC - 3′
	5′ - GGACGGACGGACTGTAACACCGACGGCGCGC - 3′

Supplementary Table S2. Primers for plasmid construction

Primer Name	Sequences
Cldn3 -510 to -504	5′ - GCATCTTTTGGGTACCTTTGCG - 3′
	5′ - CCTCCCGTACACACGTCG - 3′
Cldn3 -287 to -281	5′ - AAAAGCGCCCAAAGTGGTG - 3′
	5′ - CTTGTCCCTCTCTGCTCGG - 3′
Cldn3 +98 to +104	5′ - GTATGGAGCCGAGCCGTTAG - 3′
	5′ - TGCGGGGAGACGAGGG - 3′

Supplementary Table S3. Primers for ChIP
